# Pattern of Altered Plasma Elemental Phosphorus, Calcium, Zinc, and Iron in Alzheimer’s Disease

**DOI:** 10.1038/s41598-018-37431-8

**Published:** 2019-02-28

**Authors:** Azhaar Ashraf, Hagen Stosnach, Harold G. Parkes, Abdul Hye, John Powell, Po-Wah So, for the AddNeuroMed consortium, Hilkka Soinine, Magda Tsolaki, Bruno Vellas, Simon Lovestone, Dag Aarsland, Iwona Kloszeweska, Patrizia Mecocci, Lars-Olaf Wahland

**Affiliations:** 1 King’s College London, Department of Neuroimaging, Maurice Wohl Clinical Neuroscience Institute, Institute of Psychiatry, Psychology and Neuroscience, London, SE5 8AF UK; 2 Bruker Nano GmbH, Am Studio 2D, 12489 Berlin, Germany; 3 Institute of Cancer Research, 123, Brompton Road, London, SW7 3RP UK; 4 King’s College London, Department of Old Age Psychiatry, Maurice Wohl Clinical Neuroscience Institute, Institute of Psychiatry, Psychology and Neuroscience, London, SE5 8AF UK; 5 NIHR Biomedical Research Centre for Mental Health and Biomedical Research Unit for Dementia at South London and Maudsley NHS Foundation, London, UK; 6 King’s College London, Department of Basic and Clinical Neuroscience, Institute of Psychiatry, Psychology and Neuroscience, London, SE5 8AF UK; 7 Department of Neurology, University and University Hospital of Kuopio, Kuopio, Finland; 8 Aristole University of Thessaloniki, Thessaloniki, Greece; 9 Toulouse Gerontopole University Hospital, Universite Paul Sabatier, INSERM U 558 Toulouse, France; 10 NIHR Specialist Biomedical Research Centre for Mental Health at the South London and Maudsley NHS Foundation and King’s College London, Institute of Psychiatry, London, UK; 11 University of Oxford, Department of Psychiatry, Oxford, UK; 12 Stavanger University Hospital, Stavanger, Norway; 13 King’s College London, Institute of Psychiatry, Psychology and Neuroscience, London, UK; 14 Medical University of Lodz, Lodz, Poland; 15 Institute of Gerontology and Geriatrics, University of Perugia, Perugia, Italy; 16 Department of Neurobiology, Care Sciences and Society, Section of Clinical Geriatrics, Karolinska Institutet, Karolinska Institutet, Stockholm, Sweden

## Abstract

Metal/mineral dyshomeostasis has been implicated in the development of Alzheimer’s disease (AD). The aim of the study was to investigate the difference in absolute and percentage levels of plasma phosphorus, calcium, iron, zinc, copper, selenium in cognitively normal (CN) and AD subjects. Total reflection X-ray fluorescence (TXRF) spectroscopy was used to detect plasma metals/minerals in CN and AD subjects (n = 44 per group). TXRF detected significantly increased plasma levels of phosphorus (p = 1.33 × 10^−12^) and calcium (p = 0.025) in AD compared to CN subjects, with higher phosphorus/calcium (p = 2.55 × 10^−14^) ratio in the former. Percentage concentrations calculated for phosphorus, calcium, iron, zinc, copper, selenium by dividing the concentration of each element by the total concentration of these elements and multiplying by 100%, demonstrated phosphorus was higher in AD compared to CN subjects, while calcium, iron, zinc, copper and selenium were lower in AD subjects, with area under the curves as high as 0.937 (p = 6 × 10^−5^) computed from receiver operating curves. With exclusion of high levels of phosphorus and calcium from percentage calculations, iron levels remained low in AD whereas zinc was higher in AD, and copper and selenium levels were similar. We demonstrate altered distribution of elements in the plasma of AD subjects with high interdependencies between elemental levels and propose the potential of TXRF measurements for disease monitoring.

## Introduction

Alzheimer’s disease (AD) is the most common cause of dementia in the elderly population with a predicted incidence of 115.4 million cases by 2050. It is characterised by pathological inclusions of extracellular ß-amyloid (Aß) plaques and intracellular neurofibrillary tangles^1^. With the lack of a cure, and only mildly effective symptomatic treatments at best, there is a great impetus to understand this incurable dementia. Metal dyshomeostasis (particularly iron, zinc and copper) has recently been implicated in the development of AD^2^. These metals can accentuate plaque formation and affect tau hyperphosphorylation albeit to a lesser extent. Aberrant homeostasis of these transition elements and enhancement of protein aggregation and detrimental interactions with protein aggregates can generate oxidative stress contributing to neurodegeneration in AD^2^. Also, there has been considerable debate regarding the contribution of aluminium to AD. A common feature of neurodegenerative diseases such as AD are changes in ratios of various metals^3^.

Dyshomeostasis of other micronutrients such as calcium is also evident in the AD brain^4^. Circulating calcium and phosphorus are regulated by parathyroid hormone (PTH). In secondary hyperthyroidism, PTH levels are high, alongside normal to low calcium and a concurrent decrease in vitamin D levels^5^. High serum phosphate has been associated with increased incidence of dementia^6^ as well as changes in phosphorus/calcium ratios^7^.

AD has a well-known prodromal stage^1^ which provides a therapeutic window when pathological processes are still responsive to treatment. However, determining this prodromal stage is difficult and drives much of the current biomarker research. While cerebrospinal fluid (CSF) Aß, phosphorylated and total-tau are used as biomarkers of AD^1^, their use to monitor the prodromal phase is debatable and impractical. CSF collection is by lumbar puncture, an uncomfortable sampling method for the individual, especially the elderly and associated with morbidity, and while used to assist diagnosis, it is inappropriate for screening or repeat sampling for disease monitoring. Thus, there is the impetus towards peripheral-based biomarkers such as those in plasma which can be readily collected and repeatedly, if necessary.

Investigators have tested whether alterations of brain metals and other elements are peripherally reflected to potentially aid in diagnosis and disease monitoring^5,6,8^. No consensus has been obtained with regards to whether the peripheral concentrations of various elements are modulated in AD owing to discrepant findings. This may be attributable to plasma levels, being not only affected by AD pathogenesis, but also affected by diet, nutritional status, sex, age, etc. Additionally, the analytical method used may impact the measurements, some methods requiring sample preparation, the complexity varying between analysis methods; whether free or bound elements or their isotopes are measured, etc. A few studies do report analysis of a panel of metals by inductively-coupled plasma mass spectroscopy (ICP-MS) showing differences between cognitively normal (CN), mild cognitive impairment (MCI) and AD^2,3^.

In this study, we quantify the plasma elemental concentrations of phosphorus, calcium, iron, copper, selenium and zinc using total reflection X-ray fluorescence (TXRF) spectrometry in age- and sex-matched AD and cognitively normal (CN) individuals. TXRF measures all the isotopes of an element and detection limits can be comparable to atomic absorption spectroscopy (AAS), inductively coupled plasma optical emission spectroscopy (ICP-OES) or ICP-MS^9^. Matrix effects are minimised enabling higher sensitivity and low detection limits in the order of parts per billion. A key advantage of TXRF is the relatively simple sample preparation required compared to other techniques, contributing to its robust nature, with errors relating to pipetting small volumes rather than extraction efficiency of metals from biofluids or matrix effects. Other advantages include smaller analytical volumes needed (as low as 10 µl)^10^; simple quantification using an internal standard; and simultaneous multi-element trace analysis without bias towards expected elemental compositions^11,12^. We aim to determine whether plasma phosphorus, calcium, iron, copper, selenium and zinc; and phosphorus/calcium ratio, differentiates between CN and AD individuals and may be putative peripheral biomarkers of AD. This is the first study that we are aware of, applying TXRF to the analysis of blood for biomarker discovery in AD, and proposes a possible role of TXRF instrument in the clinical chemistry laboratory.

## Materials and Methods

### Subjects

Plasma samples from AD and CN controls were selected from the AddNeuroMed cohort^13^. Informed consent was obtained from all participants according to the Declaration of Helsinki (1991), and protocols and procedures were approved by The Joint South London and Maudsley and the Institute of Psychiatry NHS Research Ethics Committee. The diagnosis of probable AD was made according to the Diagnostic and Statistical Manual for Mental Diagnosis, fourth edition and National Institute of Neurological, Communicative Disorders and Stroke–AD and Related Disorders Association criteria, with Mini-Mental State Examination (MMSE) score of <24 for cognition. The APOE single nucleotide polymorphisms (SNPs) rs429358 and rs7412 were genotyped using Taqman SNP genotyping assays (determined by allelic discrimination assays based on fluorogenic 50 nuclease activity) and the allele inferred. In total, we examined 88 subjects: n = 44, AD; n = 44, CN; age- and sex-matched.

### Blood Collection

Blood collection was performed in the morning after an overnight fast. Venous blood was collected into lithium heparin tubes (Becton and Dickinson) and centrifuged. Plasma was separated, frozen and stored at −80 °C prior to TXRF analysis.

### TXRF of Plasma

Plasma were thawed and 20 μl of a gallium standard solution (2 mg/l, Kraft GmbH, Germany) was added to each sample volume of 20 µl. After thorough mixing, 10 μl of each solution was put onto TXRF quartz glass carriers and air dried: duplicates of each sample were performed. TXRF data was collected over 1000 s on a TXRF spectrometer (S2 PICOFOX, Bruker Nano GmbH, Germany) with a molybdenum tube excitation source operating at 50 kV/600 μA. TXRF spectra were inspected, and all elements identified, prior to deconvolution of the spectra using the PICOFOX^TM^ software. Elemental concentrations of phosphorus, calcium, iron, copper, zinc and selenium were calculated by reference to the gallium standard in each sample.

Accuracy of the TXRF measurements was validated by analysing two certified reference samples (Seronorm^TM^ serum levels 1 and 2, Sero, Norway). The lower limit of detection (with three-sigma detection) for an element e (LLD_e_) was calculated according to the following equation:

$${{\rm{L}}{\rm{L}}{\rm{D}}}_{{\rm{e}}}=3{{\rm{C}}}_{{\rm{e}}}\sqrt{{{\rm{A}}}_{{\rm{e}}}{/{\rm{A}}}_{{\rm{b}}{\rm{g}}}}$$

where C_e_ is the elemental concentration, and A_e_ and A_bg_ are the areas of the fluorescence peak for element e and the background subjacent to the fluorescence peak for e, respectively. The measurement time for the calculation of LLD_e_ was also 1000 s, the same as for the plasma samples. Each Seronorm™ standard was measured ten times.

The method detection limit (MDL) was also calculated. The MDL is defined as ‘the minimum concentration of a substance that can be measured and reported with 99% confidence that the analyte concentration is greater than zero and is determined from analysis of a sample in a given matrix containing the analyte’. The MDL_e_ for an element e was calculated (according to the U.S. Environmental Protection Agency, Title 40 Code of Federal Regulations Part 136, Appendix B, revision 1.11) using the following equation:

$${{\rm{MDL}}}_{{\rm{e}}}={{\rm{t}}}_{(n=1,{\rm{99}} \% )}\,{\rm{SD}}=2.861\,{\rm{SD}}$$

where t_(n=1,99%)_ is the students’ t-value for a 99% confidence level and a standard deviation (SD) with n − 1 degrees of freedom.

### Statistical Analysis

Data analysis was performed using SPSS IBM version 24.0. Normal distribution was checked graphically using box plots, histogram, Q-Q plots, stem and leaf diagrams and numerically using Shapiro-Wilk’s test. The following variables were square-root transformed to normality: phosphorus, copper, selenium; and calcium, iron and zinc were log transformed. Analysis of Co-Variance (ANCOVA) was used to determine elemental differences between the CN and AD groups with co-variates: diagnosis, sex, APOE status, diagnosis x sex, diagnosis x APOE, diagnosis x sex x APOE and sex x APOE. A p-value of ≤ 0.05 was considered significant. The phosphorus/calcium ratio was also calculated as in previous studies and underwent ANCOVA testing.

Absolute concentrations of metals/elements in serum have been shown to be highly variable^14^ and possibly interdependent. Thus, partial correlation analysis adjusted for age, sex, APOEε4 and diagnosis status was performed to determine interdependencies between the elements: phosphorus, calcium, iron, copper, zinc and selenium. A p-value of ≤ 0.05 was considered significant. Percentage concentrations were also calculated for phosphorus, calcium, iron, zinc, copper, selenium by dividing the concentration of each element by the total concentration of these elements and multiplying by 100%. As percentages of the elements were not normally distributed, the Mann-Whitney U test was performed to determine differences between the CN and AD, with p-value ≤ 0.05 being considered significant. As phosphorus and calcium levels were comparatively much higher than that for iron, zinc, copper and selenium, percentages for the latter elements were re-calculated with the exclusion of phosphorus and calcium and again tested for significance.

Binary logistic regression model adjusted for age, sex and APOEε4 status was used to calculate predictive probabilities of elemental percentages. These standardized predictive probabilities were then used to calculate the receiver-operator curves (ROC) and areas under the curve (AUC). For regression models, we ensured normal distribution of residuals and absence of multi-collinearity. A p-value of ≤ 0.05 was considered significant.

## Results

**Figure 1:**
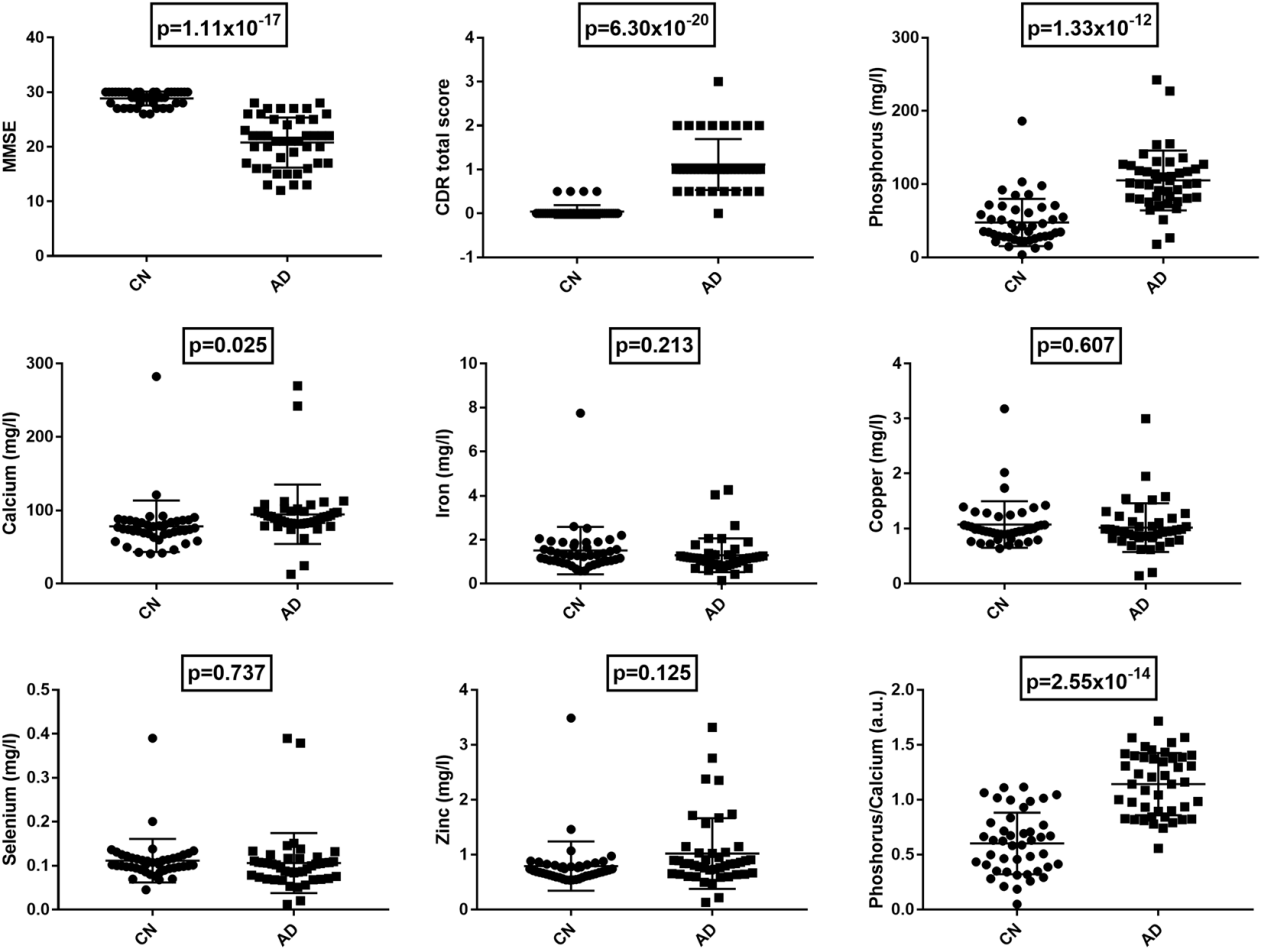
Dot plot of Total Reflectance X-Ray Fluorescence (TXRF) measurements of elements in plasma from cognitively normal (CN) and Alzheimer’s Disease (AD) subjects. P-values for each element and the phosphorus/calcium ratio are shown following normalisation of data and ANCOVA testing (see Table 1).

Demographics, clinical data, and plasma elemental concentrations for CN and AD groups are shown in Table 1 and a dot plot of the elemental concentrations shown in Fig. 1. TXRF measurements of elemental concentrations were validated using Seronorm™ Levels 1 and 2 (Table 2). Of the elements studied, selenium had the lowest abundance in the plasma and levels as low as 0.012 mg/l could be measured.

**Table 1:** Demographics, Mini-mental state examination (MMSE) and Clinical dementia rating (CDR) scores, and Total Reflection X-Ray Fluorescence (TXRF) plasma elemental measurements.

	Cognitively Normal	Alzheimer’s Disease	p-value
n	44	44	—
Age (years)	76.0 ± 5.3	77.5 ± 6.2	0.13
Females (n, %)	24 (53)	24 (57)	0.78
MMSE	28.90 ± 1.30	20.80 ± 4.60	1.11 × 10^−17^
CDR total score	0.05 ± 0.10	1.10 ± 0.60	6.30 × 10^−20^
APOEε4-positive (n, %)	17 (38)	24 (57)	0.04
Phosphorus^@^ (mg/l)	47.71 ± 31.89	105.02 ± 40.50	1.33 × 10^−12^
Calcium^#^ (mg/l)	78.44 ± 34.69	94.64 ± 39.83	0.03
Iron^#^ (mg/l)	1.51 ± 1.07	1.30 ± 0.76	0.55
Copper^@^ (mg/l)	1.08 ± 0.42	1.02 ± 0.44	0.70
Selenium^@^ (mg/l)	0.112 ± 0.049	0.106 ± 0.067	0.61
Zinc^#^ (mg/l)	0.79 ± 0.44	1.02 ± 0.64	0.93
Phosphorus/Calcium	0.60 ± 0.28	1.14 ± 0.28	0.30

Values are mean ± standard deviation. ANCOVA was performed of the transformed data adjusted for age, sex, APOE status and diagnosis and differences considered significant if p < 0.05. Data transformed to normality by square-root^@^ and log transformation^#^.

**Table 2:** Validation of Total Reflectance X-Ray Fluorescence (TXRF) measurements of plasma elemental concentrations using Seronorm™ Level 1 and 2 standards, alongside lower limit of detection (LLD, 3σ detection) and method detection limit (MDL) for each element.

Element	Seronorm™ 1				Seronorm™ 2			
	Reference~	TXRF	LLD	MDL	Reference~	TXRF	LLD	MDL
Phosphorus	70.0 ± 3.0	71.0 ± 3.5	1.8	10.0	110 ± 22	127.0 ± 5.5	1.9	30
Calcium	94.2 ± 4.4	90.0 ± 3.7	0.07	5.5	119 ± 24	123.0 ± 4.7	0.07	12
Iron	1.39 ± 0.08	1.41 ± 0.07	0.02	0.06	2.15 ± 0.43	2.07 ± 0.07	0.02	0.06
Copper	1.69 ± 0.08	1.74 ± 0.08	0.01	0.05	1.93 ± 0.39	1.88 ± 0.07	0.01	0.05
Selenium	0.107 ± 0.007	0.116 ± 0.009	0.007	0.012	0.136 ± 0.041	0.127 ± 0.012	0.007	0.006
Zinc	1.74 ± 0.07	1.72 ± 0.07	0.01	0.06	1.53 ± 0.31	1.71 ± 0.08	0.01	0.06

Values are mean ± standard deviation (mg/l). [~Reference value measured by inductive-coupled plasma-sector field mass spectrometry].

### Absolute Plasma Elemental Concentrations and Interdependencies

**Figure 2:**
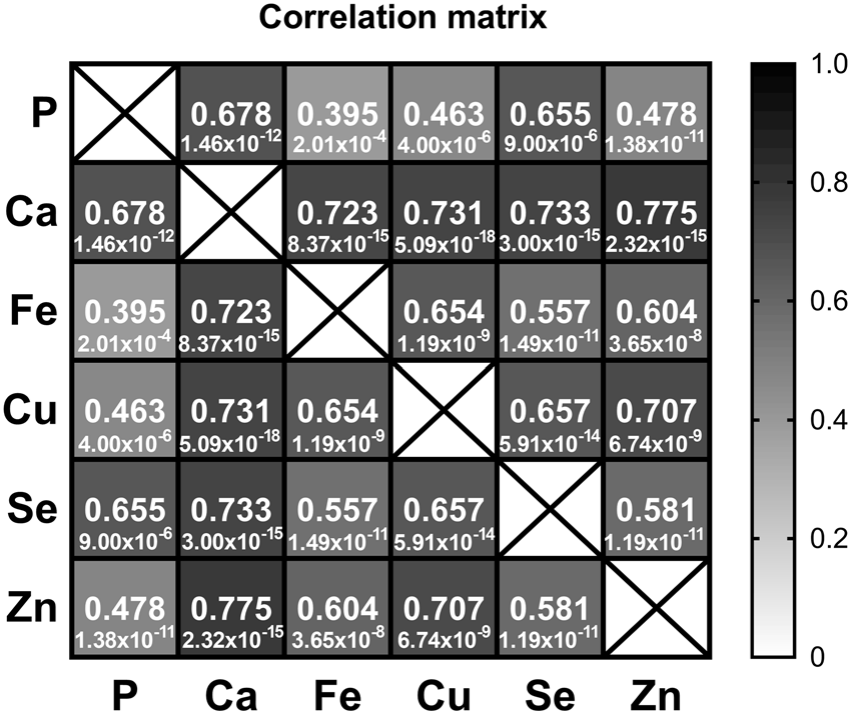
Heat map of partial correlations between the phosphorus, calcium, iron, copper, selenium and zinc (of the transformed data), controlling for age, sex, APOE status and diagnosis. (The correlation coefficient, r, is the top value in the matrix cell with the p-value below.) Phosphorus, copper and selenium have been square-root transformed, while calcium, iron and zinc were log-transformed.

Plasma phosphorus (p = 1.33 × 10^−12^) and calcium (p = 0.025) were significantly higher in AD compared to CN (Tables 1 and 3). Further, a higher phosphorus/calcium (p = 2.55 × 10^−14^) ratio was observed in AD patients (Tables 1 and 3). Absolute plasma concentrations of iron, zinc, copper and selenium were similar between the groups (Table 1). By partial correlation analysis, plasma levels of elemental phosphorus, calcium, iron, zinc, copper and selenium were shown to be strongly interdependent (Fig. 2). Hence, further statistical analysis between the CN and AD groups was performed on percentages of these elements.

**Table 3:** ANCOVA was performed of the transformed data adjusted for age, sex, APOE status and diagnosis and differences considered significant if p < 0.05.

	Age	Diagnosis	Sex	APOE	Diagnosis*Sex	Diagnosis*APOE	Diagnosis *Sex *APOE	Sex *APOE
MMSE	0.005	1.11 × 10^−17^	0.071	0.490	0.023	0.292	0.443	0.345
Phosphorous^@^	0.185	1.33 × 10^−12^	0.484	0.205	0.126	0.207	0.201	0.367
Calcium^#^	0.348	0.025	0.216	0.396	0.648	0.983	0.693	0.641
Iron^#^	0.554	0.213	0.305	0.200	0.683	0.619	0.684	0.289
Copper^@^	0.703	0.607	0.269	0.220	0.523	0.969	0.235	0.900
Selenium^@^	0.605	0.737	0.511	0.492	0.863	0.086	0.167	0.159
Zinc^#^	0.928	0.125	0.994	0.739	0.347	0.906	0.787	0.590
Phosphorus/Calcium	0.296	2.55 × 10^−14^	0.904	0.553	0.135	0.085	0.351	0.294

Data transformed to normality by square-root^@^ and log transformation^#^.

### Percentages of Plasma Elemental Concentrations

Of the six elements quantified, the percentages of phosphorus and calcium were much higher in the plasma than that for iron, zinc, copper and selenium (Table 4). Further, the percentage of phosphorus in the plasma was higher in AD than CN (p = 7.55 × 10^−11^, Table 4), whereas the percentage of calcium (p = 6.04 × 10^−11^), iron (9.85 × 10^−9^), zinc (p = 1.20 × 10^−5^), copper (p = 1.63 × 10^−10^) and selenium (p = 9.22 × 10^−12^) were lower in AD (Table 4). As phosphorus and calcium were at much higher levels than the other elements, their inclusion for the calculation of elemental percentages could potentially bias the analysis. Thus, the analysis was repeated but percentages of iron, zinc, copper and selenium were re-calculated with the exclusion of phosphorus and calcium. Again, plasma iron was significantly lower in the AD patients compared to CN subjects (Table 4; p = 0.028). However, zinc was higher in AD than CN (Table 4; p = 1.74 × 10^−4^), whereas plasma copper and selenium levels were comparable between CN and AD.

**Table 4:** Non-parametric test (Mann-Whitney U test) was performed to determine differences between plasma levels of elements between cognitively normal (CN) and AD subjects, with elements expressed as a percentage of all the elements but also with ^@^exclusion of phosphorus and calcium.

	Elemental Percentage			^@^Elemental Percentage		
	CN	AD	p-value	CN	AD	p-value
Phosphorus	34.74 ± 11.26	51.62 ± 6.50	7.55 × 10^−11^	NA	NA	NA
Calcium	62.40 ± 10.68	46.68 ± 6.19	6.04 × 10^−11^	NA	NA	NA
Iron	1.21 ± 0.48	0.64 ± 0.23	9.85 × 10^−9^	41.60 ± 8.36	37.40 ± 8.64	0.03
Copper	0.91 ± 0.36	0.51 ± 0.12	1.63 × 10^−10^	32.00 ± 6.39	30.40 ± 5.42	0.37
Selenium	0.09 ± 0.03	0.05 ± 0.01	9.22 × 10^−12^	3.37 ± 0.99	3.13 ± 1.04	0.35
Zinc	0.64 ± 0.18	0.51 ± 0.30	1.20 × 10^−5^	23.10 ± 4.52	29.00 ± 8.79	1.73 × 10^−4^

A p < 0.05 was considered significant. (NA, not applicable).

### Logistic Regression Analysis

**Figure 3:**
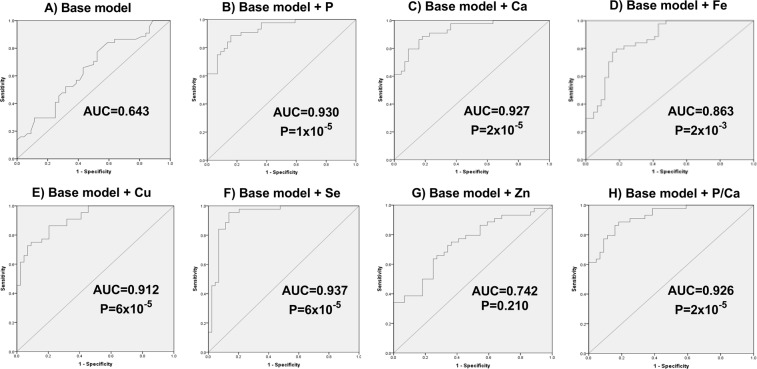
Receiver operating curves (ROC) of binary logistic regression modelling of cognitively normal (CN) and Alzheimer’s Disease (AD) subjects. Base model includes age, sex, APOEε4 genotype (**A**) and addition of percentage phosphorus (**B**); calcium (**C**); iron (**D**), copper (**E**), selenium (**F**) and zinc (**G**), and phosphorus/calcium (**H**). AUC – Area under the curve.

ROC based on the binary logistic regression model were used to determine the sensitivity of percentages of the elements in the plasma to predict CN or AD. The AUC of the base model (age, sex and APOEε4 genotype) was 0.643 (Fig. 3A), which was considerably improved by the addition of phosphorus (AUC = 0.930, p = 1.2 × 10^−5^; Fig. 3B) or calcium (AUC = 0.927, p = 1.6 × 10^−5^, Fig. 3C). The AUC also improved with addition of iron, copper or selenium (AUCs: 0.863, 0.912 and 0.937; p = 1.96 × 10^−3^, 6.00 × 10^−5^ and 6.00 × 10^−5^, respectively; Figs 2E,F and 3D). Zinc had no effect on the base model AUC (p = 0.120, Fig. 3G), while the phosphorus/calcium ratio significantly improved the AUC (0.926, p = 2 × 10^−5^, Fig. 3H).

## Discussion

We report a pattern of increased percentage of phosphorus but decreased calcium, iron, copper, zinc and selenium in the plasma from AD subjects compared to CN. High absolute levels of phosphorus and calcium were best able to discriminate between demented and non-demented cases, especially phosphorus. We demonstrate strong interdependencies between plasma levels of phosphorus, calcium, iron, copper, selenium and zinc. These findings extend and support previous studies that suggest the involvement of metal dyshomeostasis in AD but that there appears an even greater contribution from those systems regulating phosphorus and calcium metabolism.

The higher plasma phosphorus (both absolute and percentage levels) observed in AD patients in our study is consistent with a recent study showing high serum phosphate was associated with increased risk of AD, vascular dementia and Lewy Body dementia^6^. Binary logistic regression modelling demonstrated the importance of phosphorus to distinguishing AD from CN subjects. Dementia is a recognised comorbidity with chronic kidney disease in which serum phosphate is often raised^15^. Accompanying elevated plasma phosphate is an increase in fibroblast growth factor-23 (FGF-23) which itself is associated with cerebral small vessel disease that strongly drives cognitive decline and dementia^16^. Serum phosphate and FGF-23 can be increased by increasing dietary intake^17^; food, especially processed foods, commonly contain phosphate additives^18^. High phosphorus intake itself is also associated with increased cardiovascular risk and mortality^19^. Interestingly, high serum phosphate has been shown to be independently associated with the pro-inflammatory cytokine, IL-6^20^, with high phosphate intake inducing inflammation and increased serum TNFα in uremic rats^21^. Increased peripheral pro-inflammatory cytokines is characteristic of AD^22^ and high circulating phosphate may contribute to the increased inflammatory milieu in AD. Whether reducing phosphorus intake and lowering blood phosphate levels attenuates dementia remains to be investigated. Note that we have assumed that the elemental phosphorus measured by TXRF is mainly from phosphate, which is the usual form of phosphorus assessed by conventional clinical chemistry methods.

Increased plasma calcium in AD is concomitant with the increased phosphorus in our study, although decreased if expressed as a percentage of the panel of six elements which illustrates the difficulties in interpreting elemental concentrations. Alternatively, expressing the ratio of phosphorus/calcium may aid interpretation, with the ratio being higher in AD than CN. Calcium dyshomeostasis has been consistently implicated in AD^23^. Serum calcium levels are modulated by APOE, appearing to increase neuronal calcium influx to increase Aβ and associated with worse cognitive function in APOEε4 carriers, but this interaction is lost in APOEε2 carriers^24^. However, calcium changes can be mediated by non-APOE mechanisms. Plasma calcium levels are regulated by vitamin D and PTH. Hyperparathyroidism, characterised by elevated PTH, with high or elevated calcium, usually with low vitamin D levels, has been associated with impaired cognitive function and dementia^25^. Elevated blood phosphate and calcitriol (synthesized from vitamin D) deficiency may increase PTH secretion^26^ and explain the higher plasma calcium in AD observed in this study.

Lower percentage of plasma selenium in AD in this study is, consistent with previous studies. However, absolute levels were comparable between AD and CN or when comparing percentages calculated with exclusion of phosphorus and calcium, and explains the lack of consistency in alterations in blood selenium levels in AD^27^. Inclusion of selenium as a percentage of all elements studied gives the highest sensitivity and specificity to AD in this study. Selenium is often a cofactor in antioxidant molecules/pathways and the low peripheral levels in AD observed may contribute to increased susceptibility to oxidative stress, a major aspect of AD pathology. Indeed, a selenium and/or vitamin E supplementation trial had been undertaken to determine if supplementation can attenuate AD^28^. AD was not attenuated/prevented by selenium supplementation, but the study was underpowered, only involved men, and supplementation was of relatively short duration. However, selenium status has been shown to be determined by local geology and if sufficient, selenium has little impact on AD^29^.

Zinc has been reported to be decreased or unchanged in the serum and in CSF of AD patients^30^ but decreased serum zinc has been proposed to be due to ageing rather than AD^31^. Additionally, a recent ICP-MS study suggested serum zinc is increased with subjective memory complaint but decreased in MCI and AD^14^. Absolute zinc levels were comparable between CN and AD in this study but was significantly lower in the latter when normalised to the total concentrations of the panel of six elements. Conversely, zinc was higher in AD than CN if the percentage was calculated with the exclusion of phosphorus and calcium. The strong interdependences observed here and in other studies^3,32^ may contribute to the disparate findings in studies which usually only measure absolute levels of one or two metals. By considering panels of elements/metals rather than one or two of them, variations in exposure and nutrition may be accounted for somewhat (see below).

Anemia and attenuated hemoglobin levels are associated with an approximately two-fold increased risk for developing AD, establishing anemia as a risk factor for cognitive loss^8,33^. Significantly lower percentage plasma iron observed in the present study could arise due to deficient iron-loading into transferrin^8,34^ by dysfunctional ceruloplasmin, a ferroxidase that facilitates iron loading into transferrin^35^, reported in serum and CSF of AD (and PD) subjects^36^. Low peripheral iron levels may compromise hemoglobin production, leading to a significant decline in hemoglobin levels in AD^8^. Previous evidence has implicated transferrin and its receptor alleles as risk factors for AD^37^.

Copper dyshomeostasis has been implicated in AD, with reduced plasma copper and ceruloplasmin levels correlated to impaired cognitive performance in AD subjects^38^. Overexpression of APP in Tg2576 mice lead to copper deficiency, associated with impaired superoxide dismutase and ceruloplasmin prior to the appearance of amyloid neuropathology^39^. Copper supplementation has been proposed to be beneficial in AD by normalizing reduction of plasma copper^38^. In this study, we show that the percentage of copper is lower in AD than CN. However, absolute copper levels and copper percentages with the exclusion of phosphorus and calcium, were similar in CN and AD and consistent with larger published studies^40^.

Ageing is associated with a chronic inflammatory state, both peripherally and centrally and even more so, in AD^21,22^. Inflammation induces hepcidin secretion from the liver which promotes ferroportin degradation to increase iron retention in enterocytes and peripheral macrophages^41,42^. Thus, peripheral inflammation in AD may explain the decreased percentage of circulating iron levels observed in this study. Iron deficiency directly stimulates bone production and cleavage of FGF23 as well as via enhanced renal erythropoietin production^17^. Elevated peripheral inflammatory cytokines will also stimulate of FGF23 production and cleavage^16^ and as mentioned above, elevated FGF23 levels are associated with increased risks of cardiovascular disease and cognitive impairment/dementia.

Further research is required to understand the true meaning of plasma values in the context of AD. Considering the variability in exposure to environmental and/or nutritional factors (particularly in the context of malnourishment) which are significant variables, it is difficult to make precise conclusions on the liaison between the elements measured and AD. We and others^2,3^, have attempted to account for environmental factors and/or nutritional status to some degree by normalising values to a panel of elements. Changes in peripheral circulating levels of elements may reflect decreased or increased body intake/absorption and/or re-distribution between cellular and tissue compartments, e.g., increased brain accumulation. Our dataset does not include information on dietary intake of the various elements analysed in the study or their excretion from the body; any co-morbidities; and/or levels of proteins that integrate the metabolism of the elements measured. However, our cohort was age-matched, and sex balanced. There is also the concern that plasma mineral and metal measurements may reflect weight loss and malnutrition, common in AD, particularly at end stage disease. Thus, our patient cohort comprise only mild-moderate AD (MMSE ~ 20), where only 3% of individual would be expected to be malnourished^43^. We are unable to establish causality in this observational study but propose pathways worthy of further investigation in AD.

In the present study, we demonstrate altered distribution of minerals and metals in the plasma of AD and considerable interdependency in their circulating levels. Future studies should take the nutritional and environmental elemental exposure status into account to more accurately reflect the association between the elements and AD. We propose the plasma signature of high absolute levels of phosphorus and calcium, a high phosphorus/calcium ratio with a low percentage of iron may be indicative of cognitive impairment and/or AD. Further investigations are needed to determine if this pattern is common to dementia, useful for assessing the prodromal stage of AD, and/or monitoring AD progression.

## Electronic supplementary material

Supplementary Information
