# Publisher Correction: Pattern of Altered Plasma Elemental Phosphorus, Calcium, Zinc, and Iron in Alzheimer’s Disease

**DOI:** 10.1038/s41598-019-42217-7

**Published:** 2019-04-16

**Authors:** Azhaar Ashraf, Hagen Stosnach, Harold G. Parkes, Abdul Hye, John Powell, for the AddNeuroMed consortium, Hilkka Soinine, Magda Tsolaki, Bruno Vellas, Simon Lovestone, Dag Aarsland, Iwona Kloszeweska, Patrizia Mecocci, Lars-Olaf Wahland, Po-Wah So

**Affiliations:** 1 King’s College London, Department of Neuroimaging, Maurice Wohl Clinical Neuroscience Institute, Institute of Psychiatry, Psychology and Neuroscience, London, SE5 8AF UK; 2 Bruker Nano GmbH, Am Studio 2D, 12489 Berlin, Germany; 3 Institute of Cancer Research, 123, Brompton Road, London, SW7 3RP UK; 4 King’s College London, Department of Old Age Psychiatry, Maurice Wohl Clinical Neuroscience Institute, Institute of Psychiatry, Psychology and Neuroscience, London, SE5 8AF UK; 5 NIHR Biomedical Research Centre for Mental Health and Biomedical Research Unit for Dementia at South London and Maudsley NHS Foundation, London, UK; 6 King’s College London, Department of Basic and Clinical Neuroscience, Institute of Psychiatry, Psychology and Neuroscience, London, SE5 8AF UK; 7 Department of Neurology, University and University Hospital of Kuopio, Kuopio, Finland; 8 Aristole University of Thessaloniki, Thessaloniki, Greece; 9 Toulouse Gerontopole University Hospital, Universite Paul Sabatier, INSERM U 558, Toulouse, France; 10 NIHR Specialist Biomedical Research Centre for Mental Health at the South London and Maudsley NHS Foundation and King’s College London, Institute of Psychiatry, London, UK; 11 University of Oxford, Department of Psychiatry, Oxford, UK; 12 Stavanger University Hospital, Stavanger, Norway; 13 King’s College London, Institute of Psychiatry, Psychology and Neuroscience, London, UK; 14 Medical University of Lodz, Lodz, Poland; 15 Institute of Gerontology and Geriatrics, University of Perugia, Perugia, Italy; 16 Department of Neurobiology, Care Sciences and Society, Section of Clinical Geriatrics, Karolinska Institutet, Karolinska Institutet, Stockholm, Sweden

Correction to: *Scientific Reports* 10.1038/s41598-018-37431-8, published online 28 February 2019

The original version of this Article contained an error in the title of the paper, where “Pattern of Altered Plasma Elemental Phosphorus, Calcium, Zinc, and Iron in Alzheimer’s Disease” was incorrectly given as “Pattern of Altered Plasma Elemental Phosphorus, Calcium, Selenium, Iron and Copper in Alzheimer’s Disease”.

This has now been corrected in the PDF and HTML versions of the Article, and in the accompanying Supplementary Information file.

