# Supplementary material for: Pattern of Altered Plasma Elemental Phosphorus, Calcium, Zinc, and Iron in Alzheimer’s Disease
Source: Sci Rep. 2019 Feb 28;9:3147. doi: 10.1038/s41598-018-37431-8 (PMC6395674; doi:10.1038/s41598-018-37431-8)
Supplement: Supplementary file 1 — Supplementary Information [file 41598_2018_37431_MOESM1_ESM.docx]

**Pattern of Altered Plasma Elemental Phosphorus, Calcium, Zinc and Iron in Alzheimer’s Disease**

**Azhaar Ashraf^a^, Hagen Stosnach^b^, Harold G. Parkes^c^, Abdul Hye^d,e^, John Powell^f^, Simon Lovestone^g^ for the AddNeuroMed consortium, Po-Wah So^a^*.**

^a^King's College London, Department of Neuroimaging, Maurice Wohl Clinical Neuroscience Institute, Institute of Psychiatry, Psychology and Neuroscience, London, SE5 8AF, UK.

^b^Bruker Nano GmbH, Am Studio 2D, 12489 Berlin, Germany.

^c^Institute of Cancer Research, 123, Brompton Road, London, SW7 3RP, UK

^d^King’s College London, Department of Old Age Psychiatry, Maurice Wohl Clinical Neuroscience Institute, Institute of Psychiatry, Psychology and Neuroscience, London, SE5 8AF, UK

^e^NIHR Biomedical Research Centre for Mental Health and Biomedical Research Unit for Dementia at South London and Maudsley NHS Foundation, London, UK.

^f^King's College London, Department of Basic and Clinical Neuroscience, Institute of Psychiatry, Psychology and Neuroscience, London, SE5 8AF, UK.

^g^University of Oxford, Department of Psychiatry, Warneford Hospital, Oxford. OX3 7JX, UK

**Supplemental Table**

Total Reflection X-Ray Fluorescence measurements of elements in plasma of cognitively normal (CN) and Alzheimer’s Disease (AD) subjects.

| **Diagnosis** | **Phosphorus** | **Calcium** | **Iron** | **Copper** | **Selenium** | **Zinc** | **Phosphorus/Calcium** |
| --- | --- | --- | --- | --- | --- | --- | --- |
|  | **mg/l** | **mg/l** | **mg/l** | **mg/l** | **mg/l** | **mg/l** |  |
| CN | 186.22 | 282.26 | 7.744 | 3.180 | 0.390 | 3.490 | 0.660 |
| CN | 68.44 | 86.61 | 0.672 | 1.287 | 0.112 | 0.870 | 0.790 |
| CN | 97.86 | 91.92 | 1.561 | 1.047 | 0.125 | 0.880 | 1.065 |
| CN | 70.77 | 70.92 | 1.059 | 0.926 | 0.108 | 0.562 | 0.998 |
| CN | 71.72 | 77.11 | 0.816 | 0.735 | 0.101 | 0.768 | 0.930 |
| CN | 70.05 | 83.88 | 1.470 | 0.906 | 0.097 | 0.704 | 0.835 |
| CN | 60.86 | 79.14 | 1.230 | 0.724 | 0.107 | 0.612 | 0.769 |
| CN | 51.98 | 78.18 | 1.053 | 0.991 | 0.100 | 0.743 | 0.665 |
| CN | 55.22 | 88.45 | 2.201 | 1.007 | 0.138 | 1.072 | 0.624 |
| CN | 85.84 | 87.14 | 0.583 | 0.702 | 0.136 | 0.779 | 0.985 |
| CN | 84.88 | 81.20 | 1.029 | 0.902 | 0.103 | 0.809 | 1.045 |
| CN | 103.01 | 92.32 | 1.393 | 0.985 | 0.091 | 0.795 | 1.116 |
| CN | 92.22 | 90.60 | 0.911 | 1.068 | 0.111 | 0.676 | 1.018 |
| CN | 27.87 | 68.39 | 1.303 | 1.072 | 0.112 | 0.677 | 0.408 |
| CN | 14.53 | 77.89 | 1.636 | 0.762 | 0.128 | 0.828 | 0.187 |
| CN | 29.51 | 76.21 | 1.326 | 1.429 | 0.100 | 0.550 | 0.387 |
| CN | 46.20 | 68.55 | 0.992 | 0.943 | 0.095 | 0.631 | 0.674 |
| CN | 45.28 | 71.78 | 0.784 | 0.989 | 0.070 | 0.743 | 0.631 |
| CN | 51.74 | 72.24 | 1.283 | 0.989 | 0.090 | 0.781 | 0.716 |
| CN | 29.00 | 84.77 | 1.847 | 0.799 | 0.118 | 0.781 | 0.342 |
| CN | 34.25 | 74.76 | 1.128 | 0.912 | 0.102 | 0.764 | 0.458 |
| CN | 12.56 | 59.79 | 0.590 | 0.953 | 0.097 | 0.541 | 0.210 |
| CN | 28.13 | 80.36 | 1.564 | 1.065 | 0.134 | 0.744 | 0.350 |
| CN | 42.72 | 67.72 | 1.288 | 1.385 | 0.131 | 0.711 | 0.631 |
| CN | 23.18 | 72.50 | 0.924 | 1.036 | 0.103 | 0.976 | 0.320 |
| CN | 15.93 | 54.59 | 0.860 | 0.641 | 0.095 | 0.690 | 0.292 |
| CN | 25.73 | 74.11 | 1.285 | 0.956 | 0.201 | 0.655 | 0.347 |
| CN | 3.55 | 72.20 | 1.725 | 1.251 | 0.117 | 0.851 | 0.049 |
| CN | 58.36 | 57.59 | 1.493 | 0.767 | 0.125 | 0.818 | 1.013 |
| CN | 33.59 | 77.25 | 1.937 | 0.903 | 0.114 | 0.665 | 0.435 |
| CN | 36.88 | 63.31 | 1.006 | 0.970 | 0.098 | 0.614 | 0.583 |
| CN | 42.26 | 83.28 | 1.880 | 0.953 | 0.122 | 0.721 | 0.507 |
| CN | 24.31 | 86.62 | 2.058 | 0.815 | 0.119 | 0.623 | 0.281 |
| CN | 64.82 | 58.38 | 0.943 | 0.806 | 0.100 | 0.563 | 1.110 |
| CN | 21.41 | 42.92 | 1.168 | 1.053 | 0.068 | 0.543 | 0.499 |
| CN | 28.93 | 41.83 | 1.051 | 1.399 | 0.094 | 0.539 | 0.692 |
| CN | 22.43 | 46.38 | 1.166 | 1.221 | 0.115 | 0.689 | 0.484 |
| CN | 21.78 | 69.47 | 1.906 | 0.918 | 0.076 | 0.650 | 0.314 |
| CN | 35.37 | 49.94 | 1.101 | 1.307 | 0.086 | 0.638 | 0.708 |
| CN | 35.30 | 85.28 | 2.523 | 1.294 | 0.098 | 0.587 | 0.414 |
| CN | 27.45 | 40.96 | 1.380 | 0.934 | 0.080 | 0.596 | 0.670 |
| CN | 34.51 | 74.22 | 1.887 | 0.728 | 0.045 | 0.586 | 0.465 |
| CN | 31.39 | 121.35 | 2.015 | 1.739 | 0.069 | 0.886 | 0.259 |
| CN | 51.01 | 86.95 | 2.607 | 2.020 | 0.084 | 1.462 | 0.587 |
| AD | 227.22 | 269.52 | 4.261 | 2.997 | 0.390 | 2.356 | 0.843 |
| AD | 242.46 | 242.24 | 4.045 | 1.949 | 0.379 | 2.759 | 1.001 |
| AD | 141.36 | 82.40 | 1.907 | 0.837 | 0.095 | 0.599 | 1.716 |
| AD | 110.60 | 82.16 | 0.695 | 0.681 | 0.067 | 0.735 | 1.346 |
| AD | 130.46 | 83.40 | 1.066 | 1.127 | 0.145 | 0.892 | 1.564 |
| AD | 113.92 | 82.65 | 0.820 | 0.623 | 0.087 | 0.498 | 1.378 |
| AD | 135.93 | 89.37 | 0.780 | 0.962 | 0.107 | 0.656 | 1.521 |
| AD | 125.32 | 84.45 | 1.182 | 1.042 | 0.132 | 0.667 | 1.484 |
| AD | 100.41 | 87.91 | 1.266 | 0.875 | 0.104 | 1.046 | 1.142 |
| AD | 116.91 | 81.59 | 0.694 | 0.908 | 0.120 | 0.606 | 1.433 |
| AD | 118.40 | 95.76 | 1.314 | 0.976 | 0.151 | 0.765 | 1.236 |
| AD | 153.86 | 108.38 | 1.310 | 0.775 | 0.117 | 0.897 | 1.420 |
| AD | 109.99 | 91.23 | 1.160 | 0.955 | 0.125 | 0.861 | 1.206 |
| AD | 83.46 | 84.74 | 1.091 | 0.691 | 0.069 | 1.026 | 0.985 |
| AD | 17.97 | 12.95 | 0.144 | 0.143 | 0.012 | 0.132 | 1.388 |
| AD | 155.13 | 98.93 | 1.329 | 1.317 | 0.138 | 0.911 | 1.568 |
| AD | 98.93 | 86.59 | 1.392 | 0.935 | 0.111 | 0.951 | 1.143 |
| AD | 130.90 | 112.76 | 1.048 | 1.375 | 0.107 | 1.148 | 1.161 |
| AD | 101.14 | 78.01 | 0.866 | 0.905 | 0.102 | 0.598 | 1.296 |
| AD | 127.09 | 97.26 | 0.893 | 0.977 | 0.133 | 0.837 | 1.307 |
| AD | 120.68 | 86.10 | 1.136 | 0.947 | 0.108 | 0.644 | 1.402 |
| AD | 127.05 | 87.40 | 0.920 | 0.934 | 0.117 | 0.839 | 1.454 |
| AD | 101.71 | 74.23 | 1.174 | 0.826 | 0.105 | 0.645 | 1.370 |
| AD | 80.52 | 61.61 | 0.602 | 0.624 | 0.084 | 0.477 | 1.307 |
| AD | 115.07 | 83.15 | 1.216 | 0.995 | 0.086 | 0.639 | 1.384 |
| AD | 26.63 | 24.54 | 0.430 | 0.204 | 0.020 | 0.217 | 1.085 |
| AD | 117.06 | 83.24 | 2.647 | 0.794 | 0.051 | 0.891 | 1.406 |
| AD | 90.22 | 92.34 | 1.215 | 0.867 | 0.088 | 0.798 | 0.977 |
| AD | 51.35 | 91.98 | 0.960 | 1.111 | 0.068 | 0.760 | 0.558 |
| AD | 82.08 | 91.86 | 1.184 | 0.867 | 0.076 | 3.318 | 0.894 |
| AD | 79.54 | 96.54 | 1.235 | 0.993 | 0.068 | 0.721 | 0.824 |
| AD | 75.75 | 93.50 | 1.772 | 1.190 | 0.093 | 0.839 | 0.810 |
| AD | 83.44 | 98.80 | 1.571 | 1.038 | 0.109 | 0.781 | 0.845 |
| AD | 92.45 | 111.72 | 1.174 | 0.992 | 0.107 | 1.033 | 0.828 |
| AD | 64.63 | 78.92 | 0.870 | 0.755 | 0.074 | 1.148 | 0.819 |
| AD | 73.39 | 99.05 | 2.055 | 1.228 | 0.086 | 0.810 | 0.741 |
| AD | 104.84 | 112.02 | 1.242 | 1.044 | 0.070 | 0.828 | 0.936 |
| AD | 91.29 | 74.74 | 1.260 | 0.865 | 0.053 | 1.568 | 1.221 |
| AD | 66.64 | 81.28 | 1.320 | 0.873 | 0.071 | 0.589 | 0.820 |
| AD | 76.19 | 97.70 | 2.063 | 1.547 | 0.056 | 0.908 | 0.780 |
| AD | 106.78 | 102.21 | 1.022 | 1.279 | 0.103 | 1.672 | 1.045 |
| AD | 81.35 | 102.81 | 0.940 | 1.527 | 0.106 | 2.379 | 0.791 |
| AD | 100.32 | 107.59 | 0.762 | 1.586 | 0.100 | 1.716 | 0.932 |
| AD | 70.59 | 78.44 | 1.031 | 0.837 | 0.079 | 1.738 | 0.900 |
